# Diversified Morphology of Electrospun Fibers through Self-Assembly of the Strings in the Jet Developed by Flow-Induced Phase Separation

**DOI:** 10.1021/acs.langmuir.5c05253

**Published:** 2026-01-02

**Authors:** Yong-Wen Cheng, Shu-Ya Kao, Hsuan-Sheng Lin, Meng-Tzu Tsai, Hsin-Yi Lai, Pin-Hsien Lu, Chi Wang

**Affiliations:** Department of Chemical Engineering, 34912 National Cheng Kung University, Tainan 701, Taiwan, Republic of China

## Abstract

Solution electrospinning produces not only round fibers but also many unexpected features, e.g., ribbons, nets, and fibers with beads, barbs, or branches along the fiber length. These diversified features are observed in the electrospun fibers of poly­(vinyl alcohol), Nylon-6, poly­(*N*-isopropylacrylamide), ultrahigh-molecular-weight polystyrene, and ultrahigh-molecular-weight polyethylene in this study. All these unique features could be realized through our recent discoveries that flow-induced phase separation occurs in the jet to develop polymer-rich phases of hierarchical string structures with various diameters. Subsequently, lateral association (or fasciation) of the hierarchical strings ensues and creates assembled strings of different kinds, which are split from the phase-separated jet and evolve into as-spun features after solvent evaporation. Our results indicate that flow-induced phase separation in jets cannot be overlooked and is likely to unify the formation mechanism of all diversified morphologies of electrospun products.

## Graphical abstract

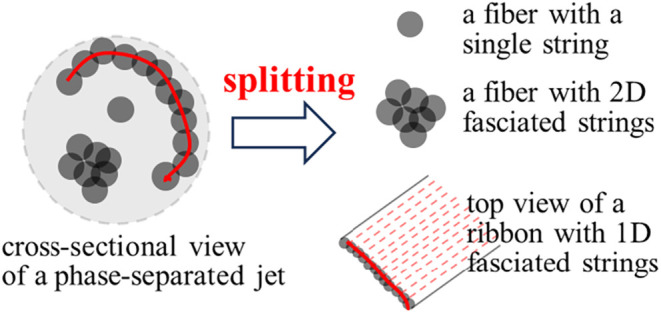

## Introduction

Solution electrospinning has become the most popular and convenient process for preparing polymer nanofibers with submicron widths for practical applications, such as filtration, composites, and scaffolds.^1–3^ The common knowledge for fiber formation is that a homogeneous one-phase solution jet, subjected to a high electric field, is continuously stretched by the induced electrical stresses to reduce jet diameter until significant solvent evaporation occurs, further impeding jet stretching and resulting in the random deposition of nanofibers on the grounded collector. Given that constant stretching of a homogeneous jet is assumed, the dried fibers must maintain a circular cross section after solvent evaporation. However, nanofibers with noncircular cross sections and net-like features are frequently produced.^4,5^ These unexpected features could be classified into two categories: The first group comprises nanofibers with variants along the fiber length, including (1) nanofibers with symmetric beads, termed “beaded fibers” by Reneker et al.;^6^ (2) nanofibers with asymmetric barbs (or spikes), denoted as “barbed fibers”;^7,8^ and (3) branched fibers, in which two separate fibers are merged to become one possessing a slightly larger (or similar) diameter.^4^ The second group exhibits 2D ribbons^4,9,10^ and 2D nets.^5,11–13^

Many hypotheses have been proposed to account for the formation of these unique features. For beaded fibers, the formation of beads is attributed to the Rayleigh instability of the thinning jet driven by surface tension.^6^ Under this assumption, a series of consecutive beads with relatively regular spacing along the fiber should be produced, and the bead size must be similar to the bead spacing. Experimental observations, however, often reveal that the bead spacing is largely irregular and is much larger than the bead diameter, implying that other sources, rather than surface tension, are involved. In fact, Rayleigh instability, being important for slightly viscous jets, could be depressed and even prohibited for highly viscoelastic jets.^14,15^ For barbed and branched fibers, some theoretical considerations have been raised for the charged jet subjected to different electrical fields for stretching: for the former, an axially stretched jet with a perturbation of surface charges along the fiber length has been considered;^8^ for the latter, a jet with undulated surface charge in the jet circumference, subjected to a cylindrical electric field, has been considered.^16^ For 2D ribbons, Reneker et al.^4^ proposed that a homogeneous jet forms a skin layer first, which is then collapsed by atmospheric pressure to become a ribbon as the solvent inside escapes. Regarding 2D nets, phase separation of polymer droplets, split from the flowing jet, was proposed by Din et al.^5,11^ According to Din’s model, the splitting droplets are homogeneous, followed by dramatic stretching by electrical forces to induce phase separation in air before reaching the collector. Despite some potential success of each hypothesis applicable to the specific feature targeted, none of these hypotheses could be applied to all the as-spun features mentioned.^4,5^ The hindrance is due to lack of comprehensive understanding of how polymer chains evolve in the flowing jet before solvent evaporation. In essence, the processing time is extremely short (several milliseconds), and the flowing jet is extremely small (diameters of several microns); these two aspects make the in situ observations of jet flow behavior difficult.

All previous hypotheses specific to the targeted features did not consider flow-induced phase separation in the flowing jet, the process of which has been validated in our recent work.^17^ We found that the extension rate of a straight jet is higher than the intrinsic relaxation rate of a given polymer solution for electrospinning,^17–19^ providing a theoretical foundation.^20,21^ Phase-separated structures of “strings” have been unambiguously discovered by rapidly freezing the internal structures of a flowing jet in liquid nitrogen or a nonsolvent bath.^22^ A new scenario of fiber formation mechanism involving flow-induced phase separation has been proposed.^18^ Remarkably, hierarchical string formation occurs along the spinline, and the cascading ordering process may produce mother strings (diameter: ∼ 400 nm) and daughter strings (diameter: ∼ 40 nm) in the jet.^18,19,22^ Some strings are thinner than the as-spun fibers, suggesting that the phase-separated structures of strings are precursor of the as-spun fibers before solvent removal.

In this study, typical polymer solutions containing either crystallizable or noncrystallizable chains are selected for electrospinning to understand how strings (and daughter strings) self-assemble to form the features of ribbons and nets, besides nanofibers. The self-assembly of the strings, via 1D and 2D aggregation in the jet cross-sectional plane, plays the key role in forming all the as-spun features mentioned above.

## Experimental Section

Five different polymer solutions were prepared for electrospinning. The temperature (*T*) of the polymer solutions was well controlled by a jacket-type heat exchanger using silicone oil as the heat carrier to ensure one-phase solution electrospinning. A stable cone-jet electrospinning mode could be achieved for hours by controlling the processing parameters of flow rate (*Q*), applied voltage (*V*), and tip-to-collector distance (*H*).

The polymer solutions used in this work are as follows: (1) Ultrahigh-molecular-weight polystyrene (UHMWPS) in dimethylformamide (DMF) solvent with a concentration of 0.5 wt %: UHMWPS pellets with a weight-average molecular weight of 2.0 × 10^6^ g/mol and a polydispersity index of 1.2 were obtained from Aldrich Co. The UHMWPS/DMF solution was electrospun at *T* = 130 °C, *Q* = 0.5 mL/h, *V* = 8.3 kV, and *H* = 14 cm. (2) Ultrahigh-molecular-weight polyethylene (UHMWPE) in *o*-dichlorobenzene (*o*-DCB) with a concentration of 0.2 wt %, together with 0.1 wt % Bu_4_NClO_4_ salt to enhance the solution conductivity: UHMWPE pellets with a weight-average molecular weight of 4.5 × 10^6^ g/mol were obtained from Aldrich Co. The UHMWPE/*o*-DCB solution was electrospun at *T* = 120 °C, *Q* = 1 mL/h, *V* = 19 kV, and *H* = 14 cm. (3) Poly­(vinyl alcohol) (PVA) in deionized water with a concentration of 7 wt %: PVA with a degree of hydrolysis of 99% and an average molecular weight of 1.66 × 10^5^ g/mol was obtained from Sigma-Aldrich Co. The PVA/water solution was electrospun at *T* = 25 °C, *Q* = 0.2 mL/h, *V* = 11 kV, and *H* = 21 cm. (4) Nylon-6 in cosolvent of formic acid (FA) and water (FA/H_2_O = 99/1 by weight) with a concentration of 15 wt %: Nylon-6 with a weight-average molecular weight of 35000 g/mol was obtained from Polysciences, Inc. The Nylon-6/FA solution was electrospun at *T* = 70 °C, *Q* = 0.3 mL/h, *V* = 21 kV, and *H* = 7 cm. (5) Poly­(*N*-isopropylacrylamide) (PNIPAM) in deionized water or DMF solvent: PNIPAM pellets with a weight-average molecular weight and polydispersity of 6.58 × 10^5^ g/mol and 1.49, respectively, were obtained from Scientific Polymer Products, Inc. The 17 wt % PNIPAM/DMF solution was electrospun at *T* = 25 °C, *Q* = 1.5 mL/h, *V* = 8.5 kV, and *H* = 14 cm; by contrast, the 11 wt % PNIPAM/water solution was electrospun at *T* = 10 °C, *Q* = 0.1 mL/h, *V* = 13 kV, and *H* = 21 cm. All the polymer solutions used in this study, including the PNIPAM/DMF solution, exhibited UCST-type phase behavior; on the contrary, the PNIPAM/water solution presented LCST-type phase behavior.^23^ Thus, careful control of solution temperature was required to ensure one-phase solution electrospinning. Because water vapor in air may trigger nonsolvent-induced phase separation^24^ in the UHMWPS/DMF and Nylon-6/FA solutions, dry nitrogen was introduced to blanket the Taylor cone from the moisture attack during electrospinning.

The morphologies of the as-spun products were examined using a scanning electron microscope (SEM, Hitachi SU8010) and a transmission electron microscope (TEM, Jeol JEM1400).

## Results and Discussion

A brief review of flow-induced phase separation in an electrospinning jet is provided in Self-Assembly of Hierarchical Strings in a Phase-Separated Jet section, according to our previous work.^17–19,22^ In Morphology of Fibers on the Grounded Collector section, fiber features obtained from six electrospinning solutions are presented and thoroughly discussed on the basis of flow-induced phase separation in the electrospinning jet.

### Self-Assembly of Hierarchical Strings in a Phase-Separated Jet

The criterion for flow-induced phase separation to occur in an electrospinning jet is that the extension rate (ε̇) of the stretched jet is sufficiently higher than the relaxation rate (*Ṙ*) of the polymer solution.^20,21^ For a given polymer solution, *Ṙ* is feasibly obtained from the rheological properties. Experimental measurement of the extension rate of a fast-flowing jet having a small diameter of several microns is difficult. Nevertheless, our recent work^17^ demonstrated the feasibility of accurate ε̇ determination of a flowing jet by a light scattering technique. On the basis of the Mie theory of cylinder scattering, the diameter of straight jets at different positions along the spinline can be determined. Jet diameter *d_j_* (*z*) decreases with increasing *z* because of electrical stretching (see Figure 1a for the *z* coordinate). After *d_j_* (*z*) is obtained, the velocity of a straight jet *v_j_* (*z*) can be calculated by 4*Q*/π*d*_j_^2^, then the extension rate is derived by a simple equation of ε̇_*z*_ (*z*) = *dv*_*j*_(*z*)/dz. The extension rate is position dependent, suggesting a dynamic stretching process by the electrical stresses due to needle-plate electrode configuration, in which the electric field *E*(*z*) for jet stretching is highest in the cone–jet transition (region I) and decays exponentially^17,25^ with increasing *z*. The magnitude of ε̇_*z*_ increases abruptly, reaches a maximum value in region I, and then decreases to a constant value in region II for the PNIPAM/DMF jet.^17^ On the contrary, the PVA/water jet exhibits a sustained growth of ε̇_*z*_ in regions I and II before jet whipping.^22^ ε̇_*z*_ derived in both jets exceeds the relaxation rate of respective polymer solutions, triggering the process of flow-induced phase separation. The phase-separated structures developed in both jets are examined after collecting the flowing jet in liquid nitrogen or a nonsolvent bath to fix its internal structure.^22,25^

**Figure 1:**
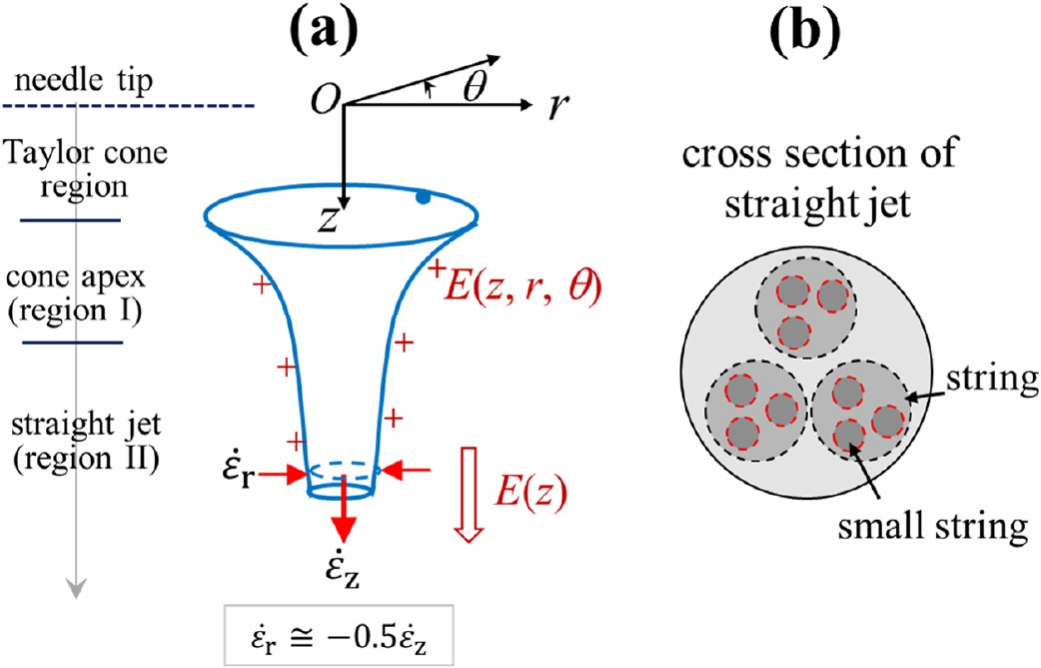
(a) Schematics of the transition region from the cone apex to straight jet during electrospinning. The cone-jet electrospinning mode is composed of a Taylor cone (not shown) pending at the needle tip (*z* = 0), followed by a cone-jet transition at the cone apex (region I) and a straight jet section with decreasing jet diameter (region II) before jet whipping. Along the spin-line, the charged jet is axially stretched by the electric stresses induced by the electric field *E*(*z*). ε̇_*z*_ and ε̇_*r*_ are the extension rate and compression rate of the jet, respectively. (b) Cross-sectional view of the straight jet, containing phase-separated domains of strings and hierarchical small strings.

The phase-separated jet is composed of long strings with various diameters.^22^ The strings are formed by a polymer-rich phase and are surrounded by a polymer-poor phase, which is nearly pure solvent. Similar phase-separated structures of strings are found in the electrospinning jets of Nylon-6/FA, PS/*o*-DCB, UHMWPS/*o*-DCB, isotactic polypropylene/*o*-DCB, and poly­(butylene terephthalate)/trifluoroacetic acid.^25^ Moreover, a hierarchical string formation (Figure 1b) is proposed to show that the (mother) string may give birth to daughter (or *small*) strings.^18,19^ The hierarchical mechanism is attributed to the following factors: (1) A mother string with an elevated polymer concentration possesses a relaxation rate (*Ṙ*_M_) lower than that of the prepared solution *Ṙ*_0_. (2) At the given external extension rate ε̇_*z*_ (>*Ṙ*_0_) and *Ṙ*_0_>*Ṙ*_M_, a secondary phase separation may subsequently occur in the mother string to give birth to daughter strings. Provided that the jet-stretching time is sufficiently long before jet drying, a tertiary phase separation in the daughter strings is likely to take place, producing granddaughter (or *fine*) strings. Coexistence of strings, *small* strings, and *fine* strings is identified in the phase-separated jet collected in liquid nitrogen.^18,22^

Because the extension rate ε̇_*z*_ in the straight jet section is large (∼500–1000 s^–1^), the induced compression rate in the radial direction ε̇_*r*_ (≈0.5 ε̇_*z*_) due to Poissonian contraction is also high, dramatically compressing the jet (Figure 2a). If ε̇_*r*_ is higher than the relaxation rate of the strings, the strings may move radially to the jet center, and lateral associations of two neighboring strings occur and induce the “fasciation process”^18^ to develop overlapped domains with a higher polymer concentration than that of the primitive strings. In this case, the osmotic pressure inside the string domain should be lower than the radial compressive stress. In other words, the extent of string fasciation depends on the relative magnitude of the imposed compressive stress and the osmotic compressibility of the liquid-like strings.^18^ In the jet cross-sectional plane, 2D fasciation of the strings creates a string bundle, composed of adhered and/or interpenetrated strings that increase its lateral dimension (Figure 2b). However, side-by-side 1D fasciation of strings may produce a ribbon-like structure with a certain width, which is relevant to the number of strings assembled.

**Figure 2:**
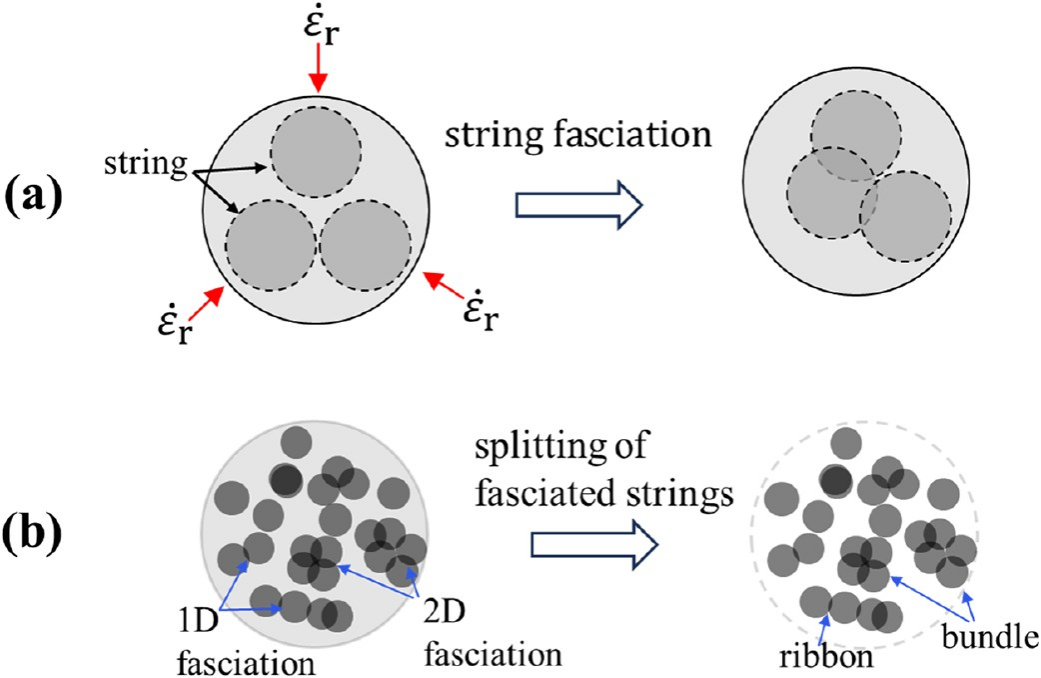
Cross-sectional view of the jet. (a) Lateral associations of the strings to form string aggregates bonded by the overlapping region. ε̇_*r*_ is the compression rate of the jet. Similar fasciation of the daughter (small) strings takes place in the string domains (not shown). (b) Splitting of the fasciated strings from the jet; these aggregates with different cross sections eventually become as-spun fibers having different widths (diameters) after solvent removal. On the cross-sectional plane of the jet, 2D fasciation of strings gives rise to string bundles, whereas 1D fasciation produces the ribbon-like structures.

### Morphology of Fibers on the Grounded Collector

Being surrounded by a polymer-poor phase, the assembled strings with enhanced stiffness are prone to being split from the jet because of the chaotic jet-whipping process.^18,22^ During solvent evaporation, the split strings having different diameters evolve into dried nanofibers on the grounded collector through a cascade of transformations: *fine string* → fibril, *small string* → microfibril, and *string* → single nanofiber along the spinline.^19^ In other words, a nanofiber is an aggregate of microfibrils, formed by the fasciation of *small strings*. Similarly, a microfibril comprises many fibrils through the fasciation of *fine strings*. The widths (or diameters) of the nanofiber, microfibril, and fibril are approximately 300–600, 50, and 10 nm, respectively.^22^

### Formation of Nanofibers with Variants along the Fiber Length

Figure 3 shows the TEM images of UHMWPS fibers collected in a carbon-coated copper grid, which is placed on top of the grounded collector. The low-magnification image in Figure 3a presents a long tapering fiber with a diameter of 470 nm at ① and a much thinner section in the regions enclosed by the dashed boxes, the images of which are enlarged in Figure 3b,c to reveal that the fiber diameter is ca. 150 and 60 nm, respectively. Nearby, another fiber is seen with a broken bulge enclosed by the dashed line at ②, spreading out its constituted components of strings after collision with the carbon film of copper grid and forming the network structures in the background. Flow-induced phase separation has been validated in the UHMWPS/*o*-DCB jet to produce fast-flowing bulges in the straight jet,^25^ the images of which are captured by high-speed videography. The bulge having a low polymer chain density mainly contains a polymer-poor solution, which is squeezed out from the highly stretched part of the jet, possessing a high polymer chain density.^26,27^

**Figure 3:**
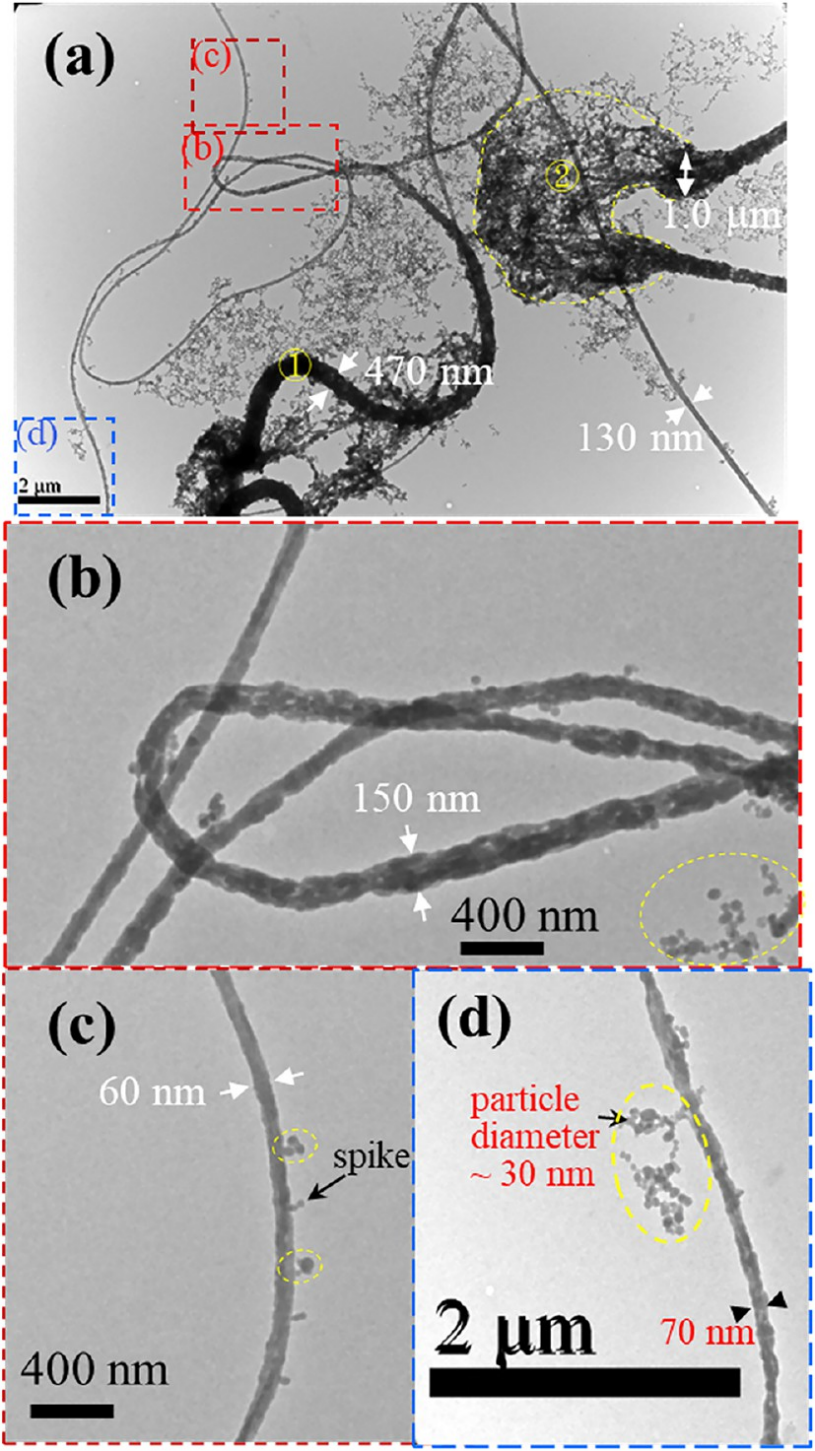
TEM images of UHMWPS fibers. The dashed boxes in (a) are enlarged to display in (b, c, and d), respectively.

In Figure 3b, the fiber surface is rough, and the internal structure of the fiber is not uniform but contains less dense parts (likely containing small voids) repeatedly appearing along the fiber length. In Figure 3c, the thinnest fiber possesses a diameter of ca. 60 nm with protruding parts, which may form a small spike, or spherical particles (beads) connected by a thin thread (as enclosed in the dashed ovals). The spherical particles connected in series by a thin thread highlighted in the dashed ovals are likely to result from (1) the relaxation of oriented chains in the strings, and (2) Rayleigh instability of the long liquid strings before solvent evaporation, driven by the surface tension. A beads-on-a-string structure is evident in Figure 3d, in which the bead has a diameter of 30 nm, and the thread diameter is ca. 15 nm. The beads-on-a-string structure is rooted from the nearby fiber with a rough surface. That is, an individual string may emanate from the string bundle and then evolves into a beads-on-a-string structure driven by the Rayleigh instability, together with chain relaxation.

The TEM images of UHMWPE fibers on the grounded collector are shown in Figures 4 and 5. In Figure 4a, two branched fibers are seen at ①, a symmetric bead at ②, and two asymmetric barbs at ③. Apparently, the bead, the barb, and the branching part along the fiber length appear simultaneously and irregularly. In Figure 4b, a fiber with a diameter of 60 nm possessing a symmetric bead having a width of 610 nm is observed; the fiber section in the dashed box is enlarged to reveal the closely packed shish–kebab structures in the inset. In Figure 4c, a fiber with a diameter of 125 nm possessing an asymmetric barb having a width of 1150 nm is identified; the fiber section nearby the barb is also enlarged in the inset to reveal the shish–kebab structure. The shish–kebab crystalline structure is readily seen in Figure 5d, which shows that the spacing between kebabs is about 15 nm. Interestingly, a thread with a diameter of 16 nm emanating from the fiber having a diameter of 160 nm is observed in Figure 5b. This structure is similar to the branched fibers shown in Figure 5c, where the branching part of the fiber having a diameter of 45 nm is likely rooted from the splitting of the main fiber with a diameter of 90 nm.

**Figure 4:**
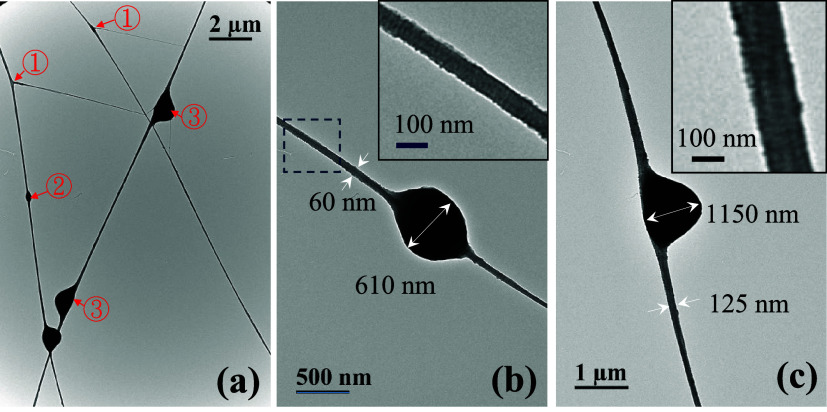
TEM images of UHMWPE fibers, (a) fibers with bead, barb and branch, (b) a beaded fiber, and (c) a barbed fiber. The insets in (b, c) are enlarged images of the fiber section to show the shish-kebab structures.

**Figure 5:**
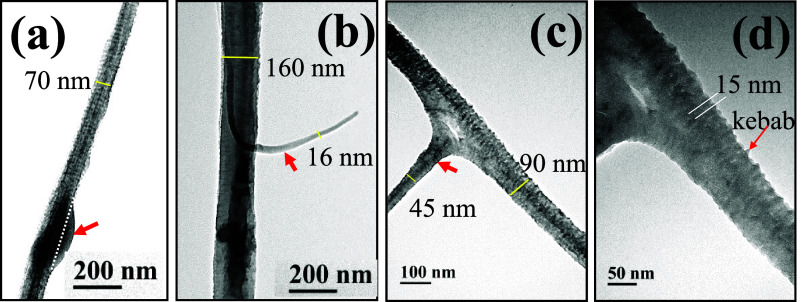
TEM images of UHMWPE fibers, (a) a fiber with an asymmetric part (red arrow) formed by the polymer-poor phase, which is squeezed out from the polymer-rich phase of strings and then dried, (b) a fiber evolving from a string with a protruding thread (red arrow), which evolves from a small string, (c) a fiber with a branching part (red arrow), and (d) enlargement of the branching part in (c). Note that the branching angle is nearly 90 °.

The findings obtained from Figures 3–5 are summarized as follows. Relatively round fibers evolve from the 2D aggregated strings. As shown in Figure 2, the string aggregation is first driven by the Poissonian contraction of the longitudinally stretched jet, followed by the lateral association of the liquid strings. Thus, string bundles having various diameters are formed in the phase-separated jet, and these bundles are surrounded by the polymer-poor matrix (nearly pure solvent). With certain rigidity, some strings are likely split out from the flowing jet and develop a secondary jet,^27^ inducing jet branching and evolving into branched fibers later after solvent removal. The splitting of the strings (and bundles) embedded in the jet most likely occurs in the jet whipping region because of the chaotic stretching process.^18^ After solvent removal from these split bundles, electrospun fibers with various diameters are produced on the grounded collector. In other words, the fasciated strings in the phase-separated jet are the precursors of the spun fibers. The diameter of individual fibers is relevant to the diameter of the individual strings and the number of strings involved in a bundle; the latter is relevant to interchain associations and the level of Poissonian contraction. Similar processes of fasciation and splitting are applicable to daughter strings and granddaughter strings. The smallest diameters detected for the UHMWPS and UHMWPE fibers are 30 and 16 nm, respectively, as shown in Figures 3d and 5b. In general, a broad distribution of fiber diameter is expected.

### Formation of Ribbons

Figure 6a shows the SEM images of fibers electrospun from 7 wt % PVA/H_2_O solution, which possesses sufficient chain entanglements to produce bead-free PVA fibers with an average fiber diameter of 275 nm.^18^ In addition to round fibers, a long plate-like aggregate of fibers with a width of 20 μm is observed. The high-magnification SEM images in Figure 6b–6d show that the plate-like feature is composed of many parallel fibers, the diameter of which is similar to that of the randomly oriented fibers in the background. Moreover, some emanating fibers are observed to protrude from the long plate-like feature. The big piece of plate-like feature is not flat but possesses a curvature. The plate-like feature composed of highly oriented fibers must be part of the jet having a diameter of ca. 10 μm.^22^ It is likely formed first by the 1D fasciation of many strings in the jet (Figure 2b) and is split out later from the flowing jet owing to its high rigidity, making it unable to be confined in the fast-flowing jet via surface tension. By contrast, the randomly oriented fibers in the background result from the splitting of individual strings, followed by the solvent evaporation. Thus, individual fibers evolve from the phase-separated structures of the strings in the jet, and 1D fasciation of the strings in the jet promotes the long plate-like feature.

**Figure 6:**
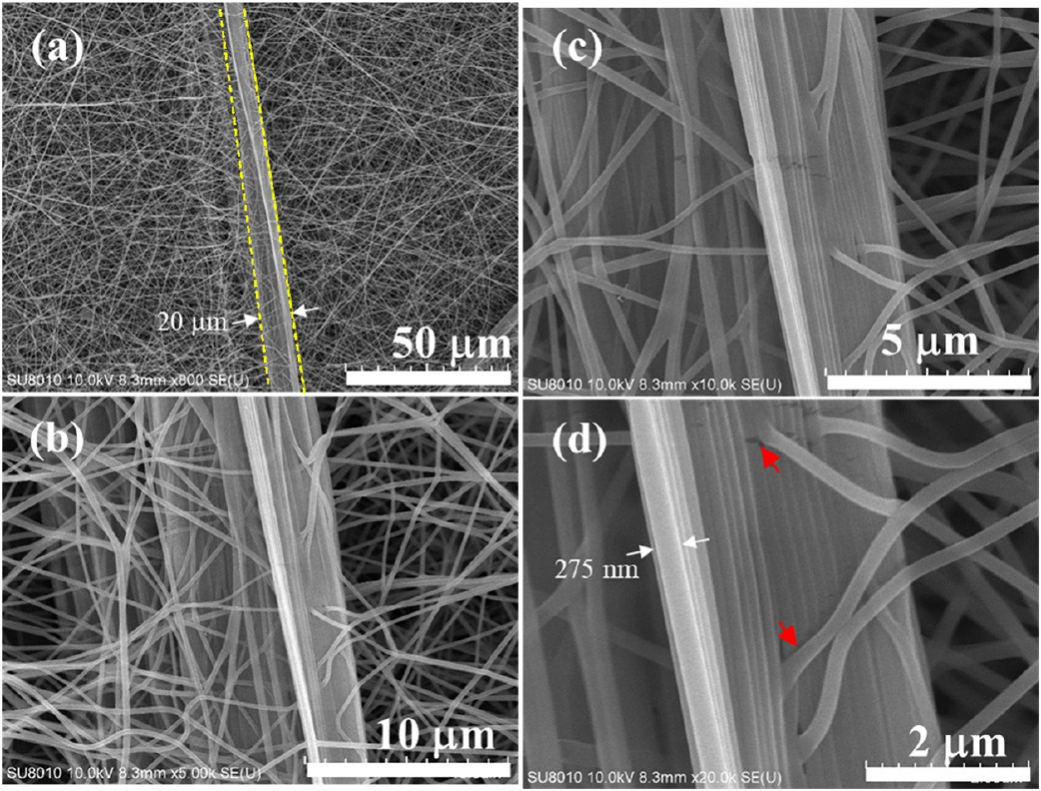
Formation of a thick PVA ribbon with curvatures. (a) SEM images of PVA fibers assembled to form a thick ribbon with a width of ca. 20 μm and a thickness of 200–275 nm, which is about the diameter of the constituted fibers. Noted that bead-free fibers are obtained in the background. (b–d) Are consecutively enlarged images of the PVA ribbon in (a) to reveal the detailed structure of thick ribbon. In (d), the emanating fibers are pointed out by the red arrows. (a) is reproduced from ref 18. Copyright 2020 American Chemical Society.

The long plate-like feature may be considered a kind of thick ribbon, composed of highly oriented *fibers*. Thin ribbons are also observed from the electrospinning of 7 wt % PVA solution, as shown in Figure 7a, exhibiting the TEM images of electrospun features collected in a carbon-coated copper grid. By examining the thin ribbon along its length from the upper-left corner, one can realize that the shape changes from a fiber-like feature with a protruding network structure in Figure 7b to a thin ribbon with a relatively uniform width of 200 nm in Figure 7c. More importantly, the thin ribbon possesses some emanating threads with diameters of 14 nm, as depicted in Figure 7d. The thin ribbon with emanating threads resembles the thick ribbon in Figure 6d, suggesting that the thin ribbon may be composed of parallel threads. In other words, the building block of the thin ribbons is the threads with a diameter of ca. 14 nm, which are closely packed laterally. The thin ribbons have evolved from the 1D fasciation of the granddaughter strings (Figure 2b), and the fasciation is so strong that the boundary between bonded strings has vanished. This phenomenon is in contrast with the transformation of the thick ribbon (Figure 6d), which evolves from the 1D fasciation of (mother) strings with mild lateral interaction, making the string boundaries still discernible.

**Figure 7:**
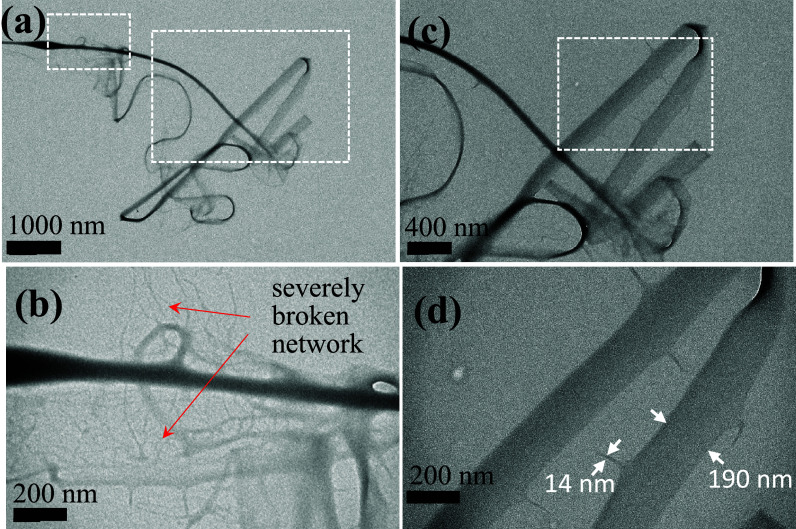
Formation of thin PVA ribbon. (a) TEM images of PVA fibers collected on carbon-coated cupper grid; the dashed boxes are enlarged to show in (b, c), and the dashed box in (c) is enlarged to show in (d).

Emanating chains from a bundle of extended chains (shish or fibril) can develop kebab lamellae, which have been proposed by Barham and Keller^28^ 4 decades ago to account for the formation of shish–kebab crystalline structures in UHMWPE microfibers. Figure 8 shows the structure similarity of the aggregates of “long lines” with three different line widths, illustrating the inevitable defects of the emanating “lines” from the “line aggregate.” The building block of flexible lines differs from one another. For the fibril in Figure 8a, the building block is the polymer chain with a diameter of ca. several Å. On the contrary, the building blocks for the thin ribbon in Figure 8b and the thick ribbon in Figure 8c are fibrils and fiber, respectively; the former has a diameter of ca. 15 nm, whereas the diameter of the latter is ca. 200 nm. The emanating chains from the fibrils (or fibril bundle) may serve as the nucleating sites for the lamellar crystallization to laterally develop the kebab crystallites. Thus, the emanating *fine* strings from the aggregates (either ribbon or bundle) may enhance the polymer crystallization in the electrospun fibers. This enhancement is confirmed in Figure 5 to show a shish–kebab crystalline structure in the UHMWPE fibers. Yoshioka et al.^29^ also identified shish–kebab structures in the electrospun nanofibers of PE having a relatively low weight-average molecular weight of 10^5^ g/mol. Using small- and wide-angle X-ray scattering, our recent work on the characterization of PVA fibers revealed the existence of fibrils in the electrospun fibers to enhance the lamellar orientation.^19^ Fibrils were also observed under an atomic force microscope in electrospun polycaprolactone fibers.^30^ Accordingly, fibrils or a fibril bundle may be formed in crystalline fibers owing to the high stretching rate in the electrospinning jet.

**Figure 8:**
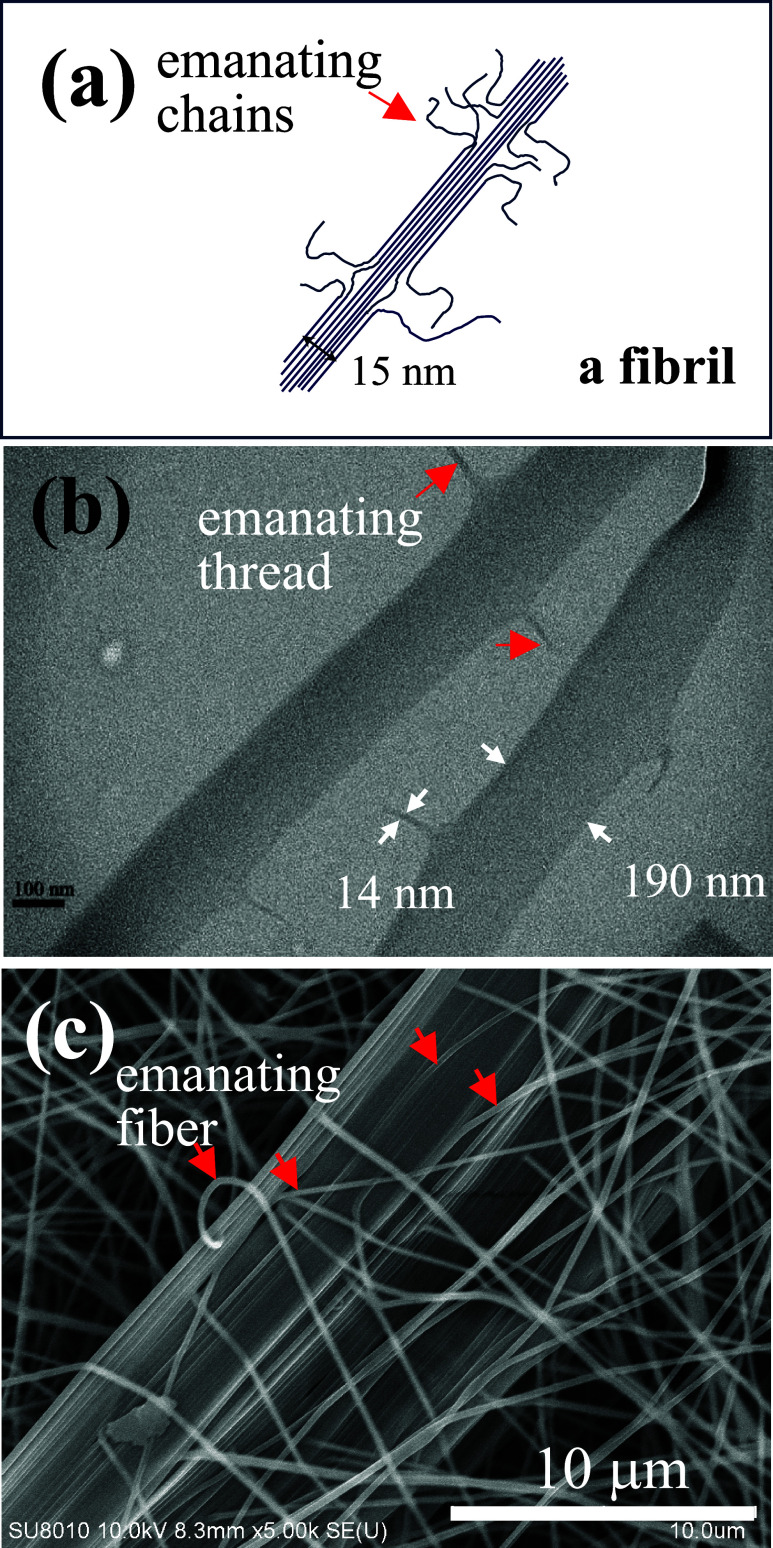
(a) Schematics of a shish (or a fibril) formed by extended chains, (b) TEM image of PVA thin ribbons formed by 1D aggregation of fibrils, and (c) SEM image of thick PVA ribbon formed by 1D aggregation of fibers. In (a–c), the emanating chains, threads, and fibers are pointed out by the red arrows to show the structure similarity despite the size difference in the constituted components. In (b), two thin ribbons evolve from 1D assembly of granddaughter strings (or fibrils) having diameters around 14 nm. In (c), a thick ribbon evolves from 1D assembly of mother strings having diameters of 200 nm. (c) is reproduced from ref 18. Copyright 2020, American Chemical Society.

Figure 9 shows the ribbons with various thicknesses electrospun from PNIPAM/H_2_O and PNIPAM/DMF solutions, regardless of the solvent used. The PNIPAM/H_2_O solution possesses LCST phase behavior, whereas the PNIPAM/DMF solution presents UCST phase behavior. The interchain association of PNIPAM macromolecules is strong because of the pending amide group, thereby readily enabling the string fasciation. In Figure 9a, the as-spun products are ribbons or branched ribbons with a wide range of width. The ribbon at ① possesses a large width of 4.3 μm and is so thin that the underneath features can still be seen. In Figure 9b–9e, the thin ribbon at ② is split from the thick ribbon (darker section, width: ∼400 nm). The thin ribbon seems to be stretched to break so that the recoiling part exhibits several folds. Interestingly, the width of the thin ribbon varies in the range of 500–850 nm, which is larger than that of the thick ribbon. On the contrary, the width of the thin ribbon at ③ is merely 300 nm.

**Figure 9:**
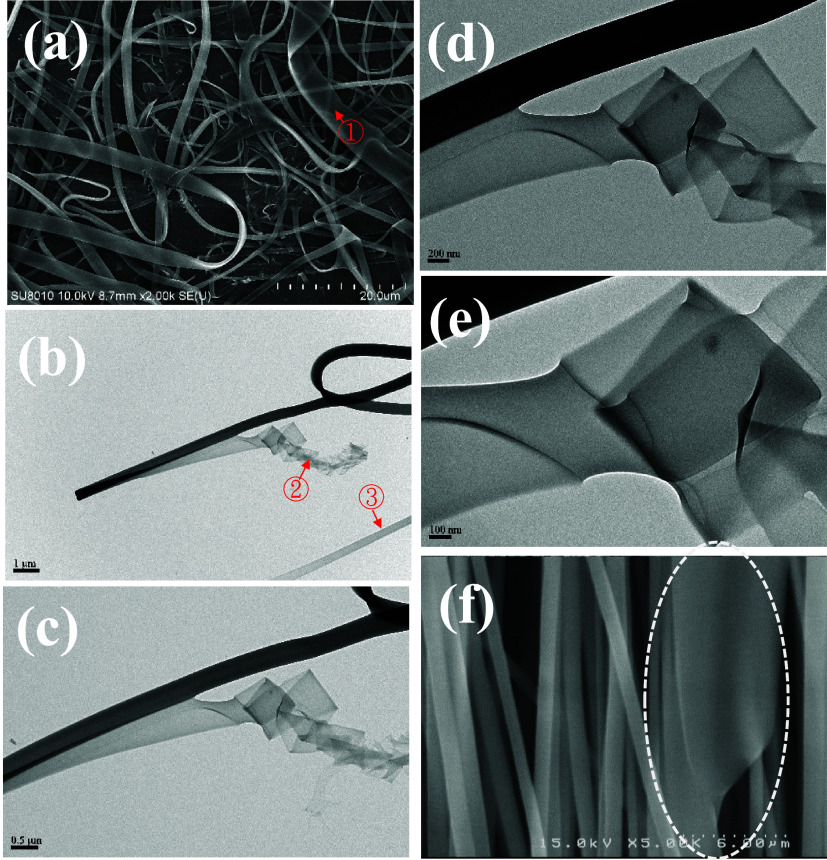
(a) SEM image and (b–e) TEM images of PNIPAM fibers electrospun from 11 wt % PNIPAM/H_2_O aqueous solution at 10 °C, and (f) SEM image of PNIPAM fibers electrospun from 17 wt % PNIPAM/DMF solution at 25 °C. The aligned fibers in (f) are obtained by using a rotating drum collector, whereas the randomly oriented fibers in (a) are collected by using a stationary steel plate. (b–e) are fibers collected in a carbon-coated cupper grid, placed on the stationary steel plate. (c–e) are consecutively enlarged images of the PNIPAM ribbon in (b) to reveal the detailed structure of thin ribbon.

Because Poissonian contraction of the jet drives the strings to move inward to facilitate 2D aggregation, as shown in Figure 2a, one may wonder about the origin of the 1D string aggregation to develop a ribbon in the jet. The driving force for this preferred 1D string aggregation is the presence of tangential velocity *v*_θ_ in the jet cross section, as depicted in Figure 10a. This additional velocity component results from the flow of a vortex (swirl), which is generated in the Taylor cone and flows intermittently into the straight jet section.^22^ The presence of swirl flow in the straight jet is validated by the observation of a twisted jet, which rapidly freezes in liquid nitrogen, followed by freeze-drying. The magnitude of *v*_θ_ may dominate the axial component *v*_*z*_ induced by the longitudinal jet stretching and the radial component *v*_r_ due to Poissonian contraction. With a large velocity gradient (i.e., ∂*v*_θ_/∂*r*), the tangential shear stress induces the strings to contact side by side to facilitate 1D fasciation, thereby creating a sheet of fascinated strings. By contrast, 2D fasciation of the strings may occur for the jet without the *v*_θ_ component, resulting from the pure jet contraction pushing the strings toward the jet center, as mentioned previously, thereby producing a string bundle.

**Figure 10:**
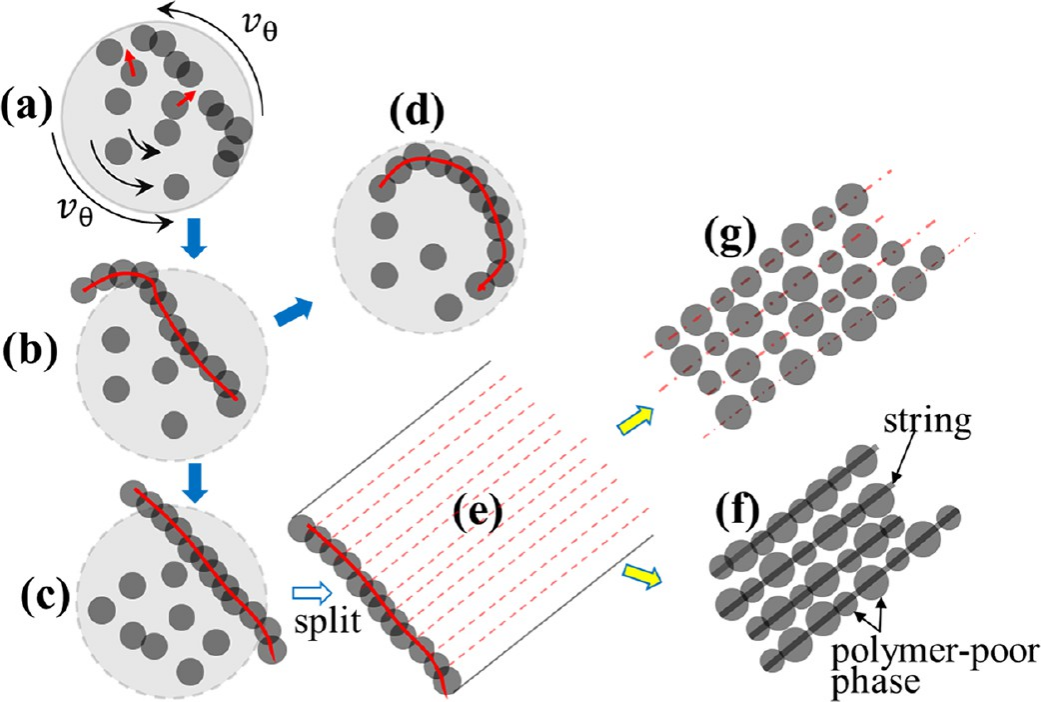
1D assembly of the strings in the phase-separated jet to form liquid ribbons. (a–d) cross-sectional view of the strings in the jet. *v*_θ_ is the tangential velocity of the jet induced by the swirl. Each gray circle denotes the cross sections of the strings. The concentration of the overlapped region of neighboring strings is enhanced. (e) a top view of the liquid ribbon in (c) which is split out from the jet. After solvent evaporation, the liquid ribbon may become a solid ribbon on the grounded collector or (f) an array of beads-on-a-string features or (g) aligned particles on the grounded collector. In other words, (e) degenerates to (f) or (g) provided that chain relaxation is fast and Rayleigh instability proceeds before the drying of liquid ribbon.

The thick ribbon with a curved edge shown in Figures 6 and 8c is generated through the process of Figure 10(a) → (b) → (d), whereas the thin ribbon shown in Figure 8b is formed by the assembly of granddaughter strings in the daughter strings through the process of Figure 10(a) → (b) → (c) → (e). The split liquid ribbon in (e) may maintain the ribbon shape during solvent evaporation if the string fasciation is strong, leading to solid ribbons deposited on the grounded collector; in this case, the thickness of solid ribbons approximates the diameter of the building blocks. If the string fasciation is weak, the liquid ribbon in (e) may degenerate to form a parallel array of beaded fibers, as illustrated in (f), or 1D aligned liquid drops shown in (g) due to chain relaxation and Rayleigh instability of the liquid strings. Whether features of (f) or (g) are formed depends on the competition between the relaxation of the string phase and solvent evaporation. Given that the former is sufficiently faster than the latter, aligned particles (feature g) will be produced on the collector (as seen in Figure 4 in ref 18.). When solvent evaporation is dominant over string relaxation, beaded fibers with regular bead spacing (feature f) will be produced. Figure 11a shows the corresponding features, like the background feature in Figure 3a, generated from the daughter strings that are randomly split from the UHMWPS mother string (i.e., the precursor of fiber). The internal structure of the UHMWPS fiber is revealed in Figure 11b, exhibiting the parallel packing of fibril bundles evolved from the daughter strings.

**Figure 11:**
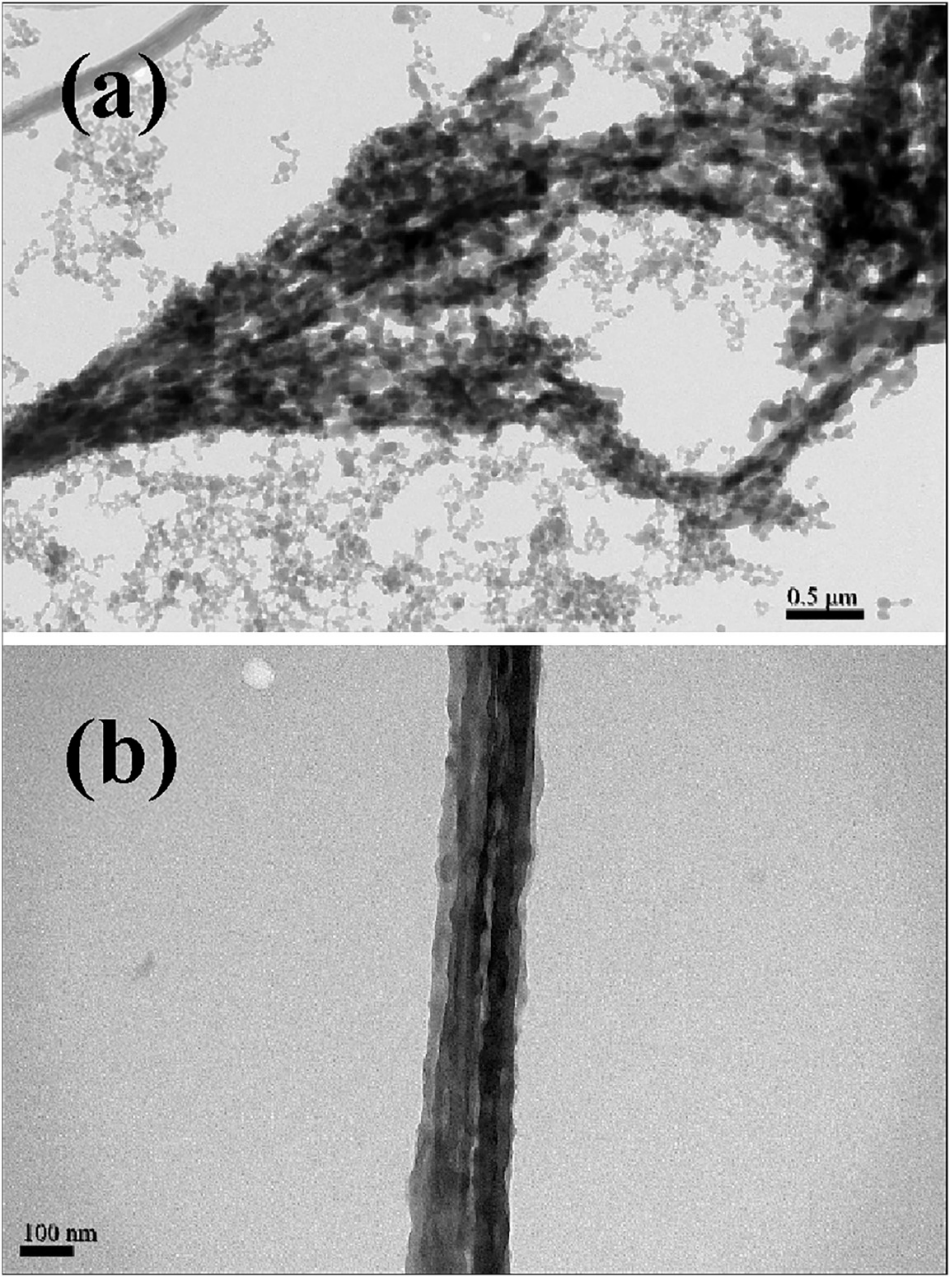
TEM images of UHMWPS fibers collected in a carbon-coated Cu grid, (a) after collision with the carbon film, a broken liquid string releases many daughter strings, which evolve to beads-on-a-string features in the background, (b) a fiber evolved from a mother string is composed of fibril bundles evolved from the daughter strings.

### Formation of Nets

Provided that sufficient interchain associations exist, e.g., PVA, PNIPAM, and Nylon-6, the level of string fasciation is significant to form a ribbon. By contrast, ribbon features are not readily seen for the PS, UHMWPS, and UHMWPE solutions despite the vortex formation in the Taylor cone. In addition to the ribbon feature, a net-like feature is produced from the Nylon-6 solution, as depicted in Figure 12a, where ① and ② are net-like structures with fine and coarse meshes, respectively, and ③ is a ribbon with 1–2 μm width. In Figure 12b, the broken part of the net is shown. In Figure 12c, a slightly broken ribbon and an unbroken ribbon are indicated at ④ and ⑤, respectively. Ribbons and regular nets (or severely damaged nets) coexist as as-spun products, suggesting the intimate formation relation between ribbons and nets. Net-like features are also seen in the as-spun product from the PVA solution, as shown in Figure 13. The ribbon has been stretched to break, followed by recoiling, to reveal that the thin ribbon is composed of very small threads, evolving from the granddaughter (or *fine*) strings. More importantly, a transition zone from the ribbon to the net with expanding lateral dimension of the ribbon is observed in Figure 13a. The assembled strings in the ribbon are torn open by the biaxial stretching at the locations where the string fasciation is weak, forming the mesh of the net. On the contrary, those strings with strong bonding with neighboring strings remain intact during the chaotic stretching, giving rise to the ribbon.

**Figure 12:**
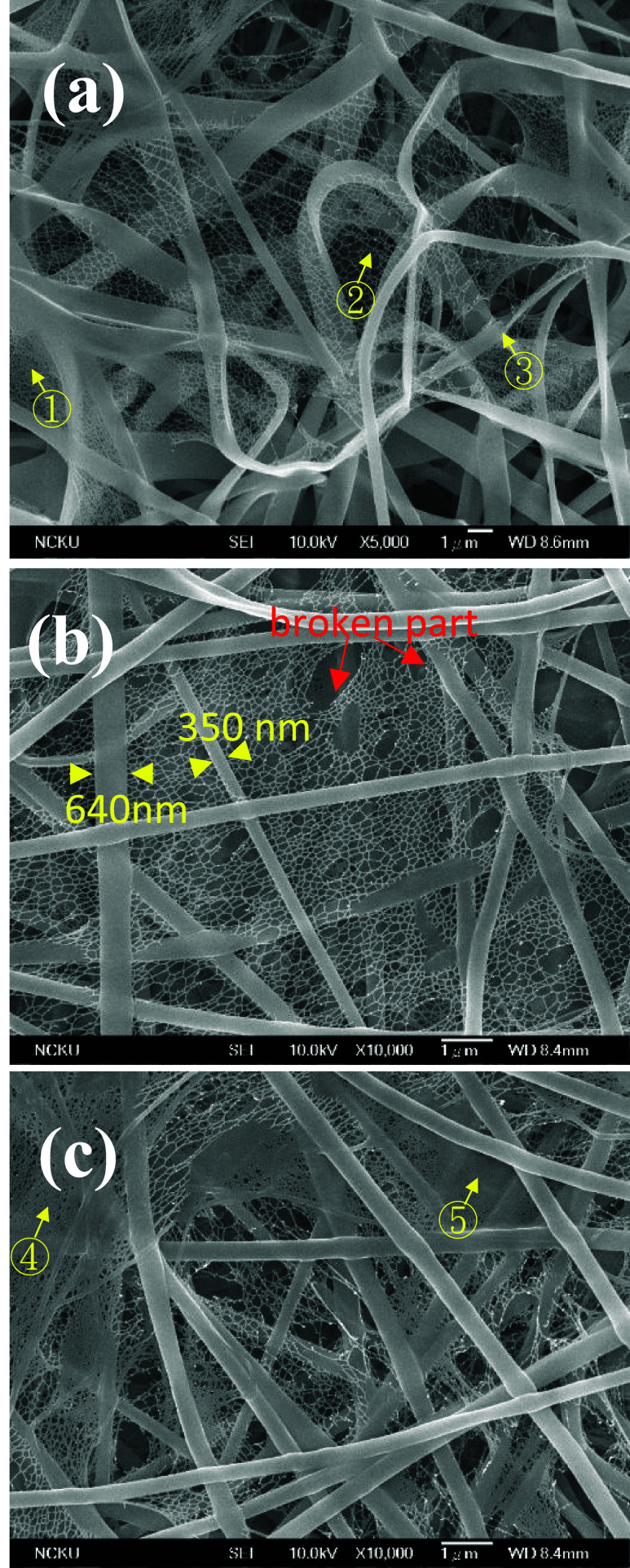
SEM image of as-spun Nylon-6 features on the grounded collector. (a) ribbons and nets, (b) nets with broken parts to show the holes having different sizes, and (c) ribbons with broken parts caused by different levels of biaxial stretching to exhibit the net-like feature.

**Figure 13:**
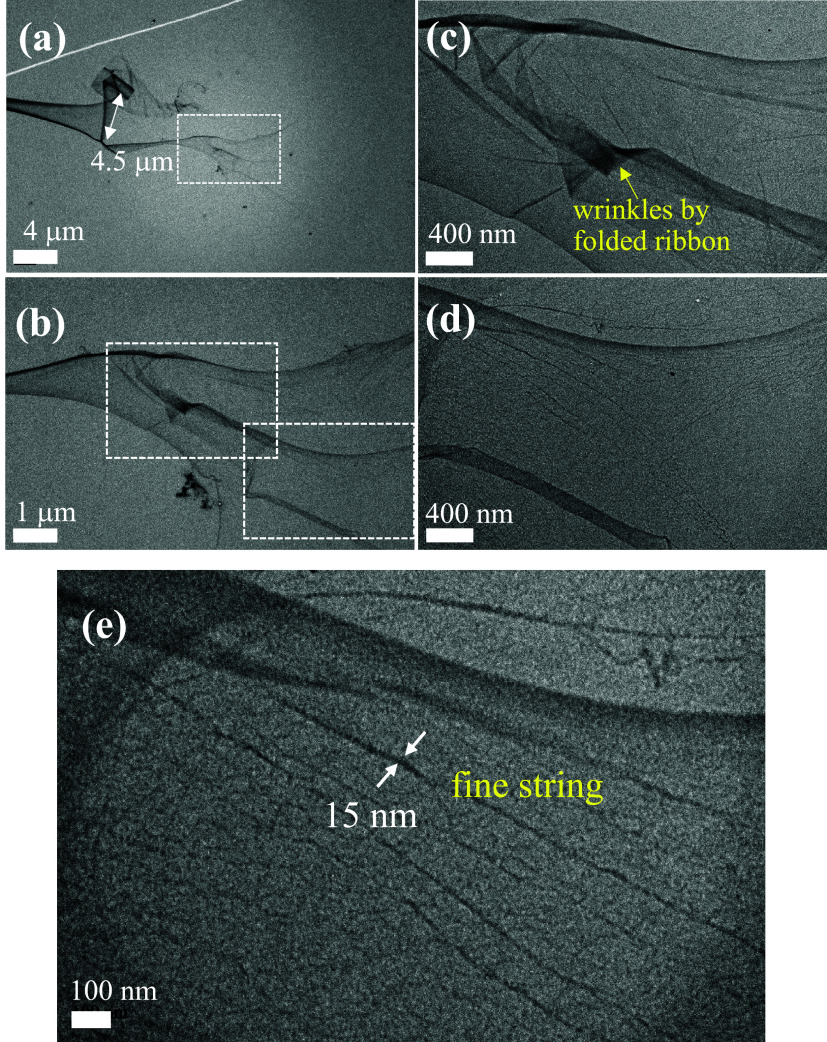
TEM images of PVA thin ribbons, which are stretched to break during electrospinning. The dashed box in (a) is enlarged in (b), and the dashed boxes in (b) are enlarged in (c, d), respectively. (e) shows the ribbon is composed of fine strings with diameter of ca. 15 nm. In (a), the white line in the upper-left corner is a long crack of carbon film.

Our hypothesis of net formation is that the liquid ribbon split out from the jet is subjected to a biaxial stretching induced by the electric field because of its 2D geometry. The liquid ribbon must be composed of parallel strings with different extents of longitudinal fasciation between strings. Those weakly bonded strings are stretched to open to form the mesh, whereas the strongly bonded strings remain to form the threads of the net. In other words, the precursor of the net is the liquid ribbon formed in the jet. A net-like feature is also likely to be produced in the jet through the intermittent lateral contacts of the parallel strings during flow. After being split out from the jet, the net-like feature composed of liquid strings evolves to a dried net with different levels of damage by biaxial stretching.

## Conclusion

Flow-induced phase separation occurs in a straight jet and form hierarchical string structures, regardless of whether crystallizable or noncrystallizable polymers are used for electrospinning.^17,22,25^ At the cross-sectional plane of the phase-separated jet, 2D aggregation of long strings creates a string bundle having a width relevant to the number of fasciated strings involved. The bundles contain emanating strings to form the branching jet, which evolves into branched or barbed fibers depending on the relaxation rate of oriented chains in the emanating strings with respect to the solvent evaporation rate. Remarkably, shish–kebab crystalline structures can be identified in UHMWPE fibers, with emanating strings as the secondary nucleating site for the kebab lamellae. For individual strings split from the flowing jet, Rayleigh instability may proceed and produce beaded fibers, given that the chain relaxation in the strings is faster than the solvent evaporation. In the extreme case, the string (or string bundle) may degenerate and evolve into aligned particles.

Under a swirl-flow field,^22^ however, 1D aggregation of long strings with a preferred linear side-by-side contact may produce a ribbon with a thickness similar to the diameter of the fascinated strings and a width relevant to the number of strings involved. Given that string fasciation is weak, longitudinal cracks of the ribbon may intermittently appear between weakly bonded strings resulting from chaotic stretching, thereby producing a net-like feature with the threads of dried strings.

The assembly of these hierarchical strings with certain rigidity cannot be confined in the spinline but is split out from the curvilinear jet and evolves into ribbons, nets, or fibers with variants along the fiber length on the grounded collector. Accordingly, all the diversified morphologies of electrospun fibers are resulting from flow-induced phase separation in the jet since the stretching rate is larger than the relaxation rate of polymer solution.^17,22^
